# Hyperhomocysteinemia and its relations to conventional risk factors for cardiovascular diseases in adult Nigerians: the REMAH study

**DOI:** 10.1186/s12872-021-01913-x

**Published:** 2021-02-18

**Authors:** Babangida S. Chori, Benjamin Danladi, Bassey A. Inyang, Michael P. Okoh, Maxwell M. Nwegbu, Adewale L. Alli, Augustine N. Odili

**Affiliations:** 1 Department of Medical Biochemistry, Faculty of Basic Medical Sciences, College of Health Sciences, University of Abuja, Abuja, Nigeria; 2 Circulatory Health Research Laboratory, Old Anatomy Block (Beside School of Nursing and Midwifery), University of Abuja Teaching Hospital, Gwagwalada, Abuja, Nigeria; 3 Department of Chemical Pathology, College of Health Sciences, Faculty of Basic Clinical Sciences, University of Abuja, Abuja, Nigeria

**Keywords:** Hyperhomocysteinemia, High-density lipoprotein, Blood pressure, Cardiovascular diseases risk factors

## Abstract

**Background:**

Evidence linking homocysteine (Hcy) with cardiovascular diseases (CVD) or its risk factors are limited in a sub-Saharan black population.

**Objective:**

We set out to evaluate the association between Hcy and hypertension and other CVD risk factors in a population of adult Nigerians.

**Methods:**

Data of 156 adults aged 18–70 years was accessed from the North Central study site of the REmoving the MAsk on Hypertension (REMAH) study. Homocysteine, blood glucose and lipid profile in whole blood/serum were measured using standard laboratory methods. Hypertension was diagnosed if average of 5 consecutive blood pressure (BP) measurements obtained using a mercury sphygmomanometer was equal to or higher than 140 systolic and/or 90 mmHg diastolic or the individual is on antihypertensive medication. Hyperhomocysteinemia (HHcy) was defined as Hcy > 10 µmol/L.

**Results:**

Of the 156 participants, 72 (43.5%) were hypertensive, of whom 18 had HHcy. Subjects with HHcy were significantly (p < 0.05) older (41.5 vs. 40.6yrs), had lower HDL-cholesterol (0.6 vs. 0.8 mmol/L) and higher systolic (145.5 vs. 126.0 mmHg) and diastolic BP (92.9 vs. 79.6 mmHg), compared to those without HHcy. Intake of alcohol and a 1 yr increase in age were respectively and significantly (p < 0.05) associated with a 1.54 and 0.10 µmol/L increase in Hcy. In a multivariable model adjusted for age, sex and body mass index, a 1 µmol/L increase in Hcy, was associated with a 1.69 mmHg and 1.34 mmHg increase in systolic and diastolic pressure (p < 0.0001) respectively; and a 0.01 mmol/L decrease in HDL-cholesterol (p < 0.05).

**Conclusion:**

HHcy occurs among hypertensive Nigerians and it is independently associated with age, HDL-cholesterol, systolic and diastolic BP.

## Introduction

Hypertension is a global health challenge increasingly responsible for most deaths worldwide. According to the Global Disease Burden survey [1], cardiovascular diseases (CVD) account for nearly 17 million deaths annually, of which more than 50% are due to hypertension related complications.

In its effort to tackle the rising burden of hypertension globally, the World Health Organisation recommends increase in physical activity, avoidance of unhealthy diet, reduction in salt intake and abstinence from tobacco use and harmful intake of alcohol [2]. Despite adopting these recommendations at population and individual levels, the burden of hypertension and other CVDs remains inadequately addressed, thus underscoring the need to explore other risk factors such as homocysteine.

Homocysteine is an intermediary metabolite of methionine whose amount in excess of 10 µmol/L (otherwise known as hyperhomocysteinemia) increases the risk of CVD in people with hypertension [3]. Compelling evidence from large-scale observational [4] and longitudinal studies [5] indicate that the risk of stroke in subjects suffering from hypertension accompanied by HHcy is higher compared to those with neither/either of the two conditions. Epidemiological data from United States of America, China and Europe indicate that among hypertensive subjects, prevalence of HHcy is 39%, 75%, and 19% respectively. Although the role of homocysteine is well-established in the development of vascular diseases [6, 7], efforts to use it as a therapeutic target of hypertension or CVD has suffered major setbacks [8, 9]. Hypothesis stimulated by recent evidence [10] indicates that the therapeutic benefit of homocysteine lowering may be affected by its interaction with other risk factors for hypertension or CVD, hence may vary from population to population. In Nigeria, 38% of its adult population are hypertensive, of whom control of blood pressure is achieved only in 12% [11] and knowledge of the burden of hyperhomocysteinemia is uncertain. The present study was aimed at evaluating the burden of hyperhomocysteinemia among adult Nigerians and also to assess the relation of homocysteine with hypertension and established risk factors for CVD.

## Methods

### Sampling/study participants

Participants of the current study were of a sub-sample of adult Nigerians aged 18 years and above that participated in the Removing the Mask on Hypertension (REMAH) Study [11, 12]. REMAH was a nationwide study that surveyed hypertension and its risk factors in the six geopolitical zones of Nigeria. From each zone, subjects were drawn from two communities of a state including Anambra, Akwa-Ibom, Oyo, Zamfara, Gombe and FCT-Abuja using a multistaged sampling technique. Communities were sensitized and mobilised, following which houses were systematically numbered, randomly selected and visited to issue invitations to eligible adults aged 18 years and above. Physical examinations were conducted on consenting participants further to which blood samples were collected for biochemical examinations. REMAH strictly complied with the Helsinki Guidelines [13] for conducting research on human subjects. For the purpose of this homocysteine sub-study, we used a prevalence rate of 51% (from a previous survey of HHcy among adult Nigerians) [14], a margin of error of 8% and static confidence level of 95%, to determine the sample size sufficiently powered for this study. We estimated a total of 150 subjects. Using a systematic random technique, we selected 156 subjects from the 653 subjects of the FCT-Abuja study site whose serial number was a multiple of 4. Subjects were excluded if they reported current use of folic acid, vitamin B6, vitamin B12 supplementation or any lipid lowering medications.

### Biochemical examination

Non-fasting blood samples were collected and the resulting sera stored at − 20 °C until analysis. Homocysteine was estimated from blood serum using the sandwich enzyme-linked immunoabsorbent (ELISA) assay method of Engvall and Perlmann [15]. Random blood glucose was measured from whole blood using accuchek glucometer®. Other biochemical analytes including total cholesterol, high density lipoprotein cholesterol (HDL-C), low density lipoprotein cholesterol (LDL-C) and triacylglycerol (TAG) were estimated at the Circulatory Health Research Laboratory using Landwind c100® autochemistry analyser. Analyses were done using ready-to-use reagents obtained from Beijing Strong Biotechnologies incorporation, Haidan District, Beijing China in accordance with manufacturer’s guide.

#### Blood pressure measurement

Blood pressure was obtained by trained observers using Accoson® mercury sphygmomanometer. Observers received training on blood pressure measurement using the British Hypertension Society Blood Pressure Measurement Educational Video [16]. They measured brachial blood pressure by auscultation of the Korotkoff sounds at the non-dominant arm in accordance with the 2013 guidelines of the European Society of Hypertension (ESH)/European Society of Cardiology (ESC) [17]. Participants were allowed to sit and rest for 5 min after which 5 BP readings are obtained at an interval of 30 to 60 s. Systolic and phase V diastolic blood pressures are determined to the nearest 2 mm Hg. Standard cuffs with 12 × 24 cm inflatable bladders are used for subjects whose arm circumference is less than 31 cm, and 15 × 35 cm for those with arm circumference greater than 31 cm.

A participant’s clinic BP is an average of the 5 consecutive BP measurements.

### Definition of terms

Hypertension was defined according to the 2013 ESH/ESC guidelines [17] as systolic blood pressure ≥ 140 mm Hg and/or diastolic blood pressure ≥ 90 mm Hg and/or self-reported treatment of hypertension using antihypertensive medications. Hyperhomocysteinemia was defined according to the American Heart Association/ American Stroke Association Council on Stroke as a serum homocysteine greater than 10 µmol/L [18].

#### Other measurements

Field officers measured weight and height in kilogram and centimeter respectively, using a stadiometer. Waist and hip circumference were measured in centimeter using a non-stretchable tape. They also administered a modified WHO STEPs Questionnaire to collect information on relevant medical history, alcohol and cigarette consumption, and intake of medications.

### Database management and statistical analysis

We used SAS software version 9.4 (SAS Institute, Cary, NC) for data management and statistical analysis. We used mean and standard deviation to respectively measure the central tendency and dispersion of continuous variables. Differences of means between binary groups were tested using student t-test. We used numbers and percentages to express categorical variables and differences in percentages were tested using chi-square. We regressed serum homocysteine on biochemical, anthropometric, socio-demographic and physiological characteristics to determine their associations. We further tested for Independent association of serum homocysteine with other variables in a multivariable model adjusted for age, sex and BMI. The null hypothesis was rejected for 2-sided values of P < 0.05.

## Results

### Characteristics of study participants

Table 1 shows the baseline characteristics of subjects stratified based on their hyperhomocysteinemia status. A total of 156 subjects with an average age of 41.5 years were included in this study. Of the entire subjects, 72 (46.2%) were women, 84 (53.8%) were men and 18 (11.5%) had hyperhomocysteinemia. In comparison to subjects without hyperhomocysteinemia, subjects with hyperhomocysteinemia had significantly higher (p < 0.05) mean age (49.0 vs. 35.0 yrs), higher mean systolic blood pressure (145.5 vs. 126.0 mmHg), higher mean diastolic blood pressure (92.9 vs. 79.6 mmHg) and lower HDL-C (0.6 vs. 0.8 mmol/L).

**Table 1:** Baseline characteristics of participants

	Overall	Hcy < 10 µmol/L	Hcy ≥10 µmol/L	p-Value
Number (%)	156	138 (88.5)	18(11.5)	
Women	72(46.2)	65(90.3)	7(9.7)	0.5109
Men	84(53.8)	73(86.9)	11(13.1)	
*Mean ± SD*				
Age	41.5 ± 13.6	40.6 ± 12.9	48.0 ± 17.0	0.0247
BMI, Kg/m^2^	25.5 ± 5.2	25.5 ± 5.1	25.4 ± 6.4	0.9674
WHR	0.89 ± 0.09	0.89 ± 0.09	0.89 ± 0.07	0.9957
Systolic BP	128.2 ± 20.9	126.0 ± 20.0	145.5 ± 20.3	< 0.0001
Diastolic BP	81.1 ± 13.7	79.6 ± 13.2	92.9 ± 11.6	< 0.0001
Random blood glucose	5.98 ± 2.08	6.0 ± 2.0	5.9 ± 2.4	0.7735
HDL-C, mmol/L	0.78 ± 0.38	0.8 ± 0.4	0.6 ± 0.4	0.0103
LDL-C, mmol/L	2.04 ± 0.98	2.0 ± 1.0	2.2 ± 1.0	0.5124
TAG, mmol/L	1.42 ± 0.68	1.4 ± 0.7	1.5 ± 0.4	0.6315
Total Cholesterol, mmol/L	3.47 ± 1.06	3.5 ± 1.0	3.4 ± 1.0	0.8738

### Prevalence of hyperhomocysteinemia

**Fig. 1:**
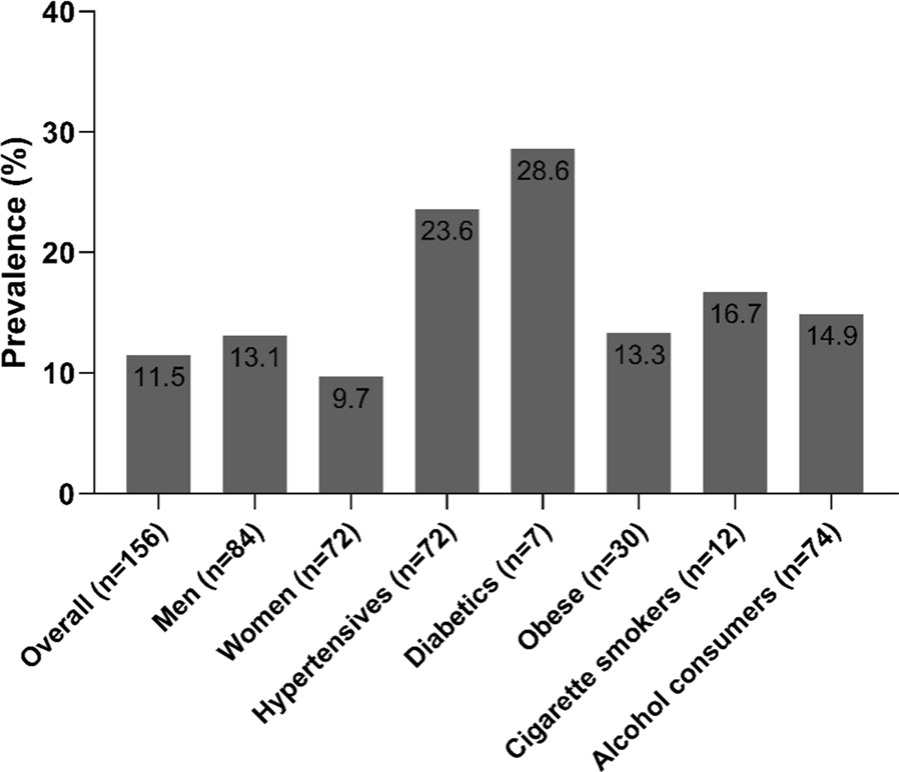
Prevalence of hyperhomocysteinemia

Hyperhomocysteinemia (HHcy) was prevalent in 11.5% of the overall subjects; 13.1% in men, 9.7% in women and 23.6% in subjects with hypertension (Fig. 1).

### Association of homocysteine with sociodemographic characteristics

According to Table 2, homocysteine (Hcy) correlated positively with age and alcohol intake. Intake of alcohol and a 1 year increase in age were significantly (p < 0.05) associated with 1.54 and 0.10 µmol/L increase in Hcy respectively. In a multivariable model, mutually adjusted for age, sex and BMI, the relationship remained the same only for age (Table 3).

**Table 2:** Univariate linear regression analysis of association of homocysteine with sociodemographic, anthropometric, biochemical and physiological characteristics

	β value	P value
*Socio-demographic*		
Age	0.097	0.0005
Sex	1.015	0.1856
Cigarette smoking	1.755	0.2215
Alcohol intake	1.542	0.0442
Body Mass Index	0.083	0.3449
Waist-hip ratio	0.002	0.3200
*Biochemical*		
LDL	0.007	0.6824
HDL-C	− 0.015	0.0166
TAG	0.026	0.0252
Total Cholesterol	0.003	0.8568
Blood Glucose	0.044	0.2148
*Physiological*		
Systolic BP	2.220	< 0.0001
Diastolic BP	1.564	< 0.0001
Pulse rate	0.353	0.1179

**Table 3:** Multivariable linear regression analysis of association of homocysteine with socio-demographic, biochemical and physiological characteristics

Characteristics	*β value	P value
*Sociodemographic*		
Age^a^	0.095	0.0008
Alcohol Intake^b^	1.091	0.1488
*Biochemical*		
HDL-C^c^	− 0.015	0.0301
TAG^c^	0.013	0.2651
*Physiological*		
Systolic BP^c^	1.691	< 0.0001
Diastolic BP^c^	1.299	< 0.0001

^a^Adjusted for sex, alcohol intake, cigarette smoking and BMI

^b^Adjusted for age, sex, cigarette smoking and BMI

^c^Adjusted for age, sex, cigarette smoking, alcohol intake and BMI

### Association of homocysteine with biochemical characteristics

A 1 µmol/L increase in Hcy associated positively with triacylglycerol (β = 0.03, p = 0.0252) and negatively with HDL-C (β = -0.02, p = 0.0166). The relationship between Hcy and HDL-C remained the same after adjustment for age, sex, BMI, intake of alcohol and cigarette smoking.

### Association of homocysteine with blood pressure

Increasing Hcy by 1 µmol/L was associated with a corresponding increase of 2.2 mmHg in systolic blood pressure (SBP) and 1.7 mmHg in diastolic blood pressure (DBP). Adjustment for age, sex, BMI, intake of alcohol and cigarette smoking did not alter the association of Hcy and systolic or diastolic BP.

## Discussion

The main finding of this study was that hyperhomocysteinemia (HHcy) occurred in nearly one-eight of the overall subjects and one-quarter of the hypertensive subjects. Homocysteine (Hcy) independently associated with age, high-density lipoprotein cholesterol (HDL-C), systolic and diastolic blood pressure.

Due to the use of different cut-offs for HHcy in different studies, it is difficult to discuss our findings in the light of those of other studies. Our decision to use the 10 µmol/L threshold was driven by expert opinions [19] which suggest that commencement of Hcy reduction at this level is more beneficial than at higher levels. Previous studies of HHcy among hypertensive Nigerians were hospital-based and were done using a threshold of 15 µmol/L [14, 20]. The prevalence of HHcy among hypertensive subjects in the current study (23.6%) was strikingly lower than the 56.0% [20] and 98.3% [14] reported by the previous studies. Although the cause of this wide difference is unknown, we hypothesise that difference in study participants may be a plausible explanation bearing that subjects of the current study were drawn from an apparently healthy semi-urban general population with a socio-economic status that could afford them a vitamin-rich diet well known to protect against HHcy. This hypothesis is based on evidences from large scale observational studies [21, 22] which have shown that due to limited financial resources, rural compared to urban dwellers tend to prioritise high-caloric foods over fruits and vegetables given that the former is relatively cheaper. The influence of vitamin-deficient diet on plasma Hcy, possibly explained by low socio-economic status, may also be observed in other surveys involving rural-dwelling Chinese subjects which recorded HHcy in more than 75% of the hypertensive cohorts [23, 24]. Chang and colleagues reported that among 3392 low-income hypertensive subjects who resided in a rural area of North-Eastern China, more than 85% had HHcy and an average diet score of 2.2 which is suggestive of a diet pattern low in vegetables. Vitamin B6, B12 and folic acid are essential requirements of Hcy metabolism which when deficient, precipitates an elevation in plasma Hcy. Other reports across the globe have shown that among hypertensive populations, HHcy occurs 39.4% in the United States of America (USA) [5] and 18.7% in Europe [25]. In addition to diet, other factors directly influencing plasma Hcy concentration are enzymes responsible for Hcy metabolism such as methylene tetrahydrofolate reductase (MTHFR) [26] and cystathionine beta synthase (CBS) [27] whose defective or variant forms could be involved in geographical/racial phenotypic variation of HHcy. In the Hordaland homocysteine study including over 18,000 adult Norwegians aged at least 40 years, low folate status accompanied by Hcy > 40 µmol/L and attributable to the MTHFR 677C > T polymorphism was prevalent in over 78% of the study participants [28]. In some parts of North America such as Canada, the MTHFR 677C > T polymorphism accounts for elevated Hcy in almost 40% of Franco-Canadians and ranges between 5 and 15% of the general Canadian population [29].

The relation of Hcy with lipid fractions remains largely inconsistent across various observational and experimental studies [27, 30, 31]. Nevertheless, our findings were in concordance with some earlier reports. Evidence from a cross-sectional survey of 4,660 Chinese subjects in Shijingshan district of Beijing [31], showed that increasing Hcy was associated with an increased risk of low HDL-C (AOR = 1.41, 95% CI 1.14–1.73) and hypertriglyceridemia (AOR = 1.29, 95% CI 1.10–1.52). In 300 Indian patients with coronary heart disease, Mahalle and colleagues [32] reported that Hcy was directly proportional to TAG and VLDL but inversely proportional to HDL-C. Experimental studies involving the use of double knock-out mice model (CBS−/−/apo-E−/−) with reduced CBS activity showed lower HDL-C (19.9md/dl vs. 24.4 mg/dl) and higher Hcy (210 µM vs. 3.8 µM) when compared with single knock-out (CBS + / + /apo-E−/−) [27]. The mechanism underlying the unfavourable association of Hcy with lipid fractions involves various transcription factors and enzymes of lipid metabolism including peroxisome proliferator-activated receptor alpha (PPARα), lecithin-cholesterol acyl transferase (LCAT) and sterol regulatory element binding proteins (SREBPs). Hcy interrupts the transcriptional activities of PPARα, thereby down regulating the production of apo-AI which is an indispensable component of HDL-C production and biological activity [33]. It is also most probable Hcy interruption/disruption plays some significant roles in the co-regulation of some downstream retrotransposons such as transposable elements (TEs), repetitive sequences in mammalian genomes, which have been implicated in many of the same conditions for which PPARα agonists are therapeutic including neuro-degeneration, schizophrenia, and drug addiction. Furthermore, Hcy also interferes with HDL-C production by inhibiting the activity of LCAT [34, 35] which is essential for reverse cholesterol transport. Conversely, Hcy enhances the expression of SREBPs, a membrane bound transcriptional factor of the endoplasmic reticulum which encodes enzymes of cholesterol/TAG metabolism and uptake [36]. These biochemical mechanisms explaining the interaction between Hcy and lipid metabolism are consistent with the results of this study.

Though there are mixed views concerning the link between Hcy and blood pressure [10, 28, 37–39], various mechanisms in support of a positive association have been put forward [6, 40, 41]. Under conditions of HHcy, uptake and synthesis of cysteine (an essential precursor of gluthathione) is reduced and antioxidant potential compromised thus leading to oxidative stress [42]. Additionally, Hcy is also metabolised into Hcy-thiolactone, a reactive specie which when bound to collagen, inhibits its activity [7]. The resultant effect of these is endothelial damage, endothelium-myocyte uncoupling, hindered passage of nitric oxide through the matrix barrier, poor vasodilation, diastolic dysfunction and high blood pressure [6, 40]. Other possible mechanisms include increased arterial stiffness, increased arteriolar constriction, increased sodium reabsorption, poor elastic vascular wall and smooth cell proliferation [6]. In a case-controlled study involving 2,615 Chinese subjects, Yang and colleagues [10] observed a strong positive correlation between Hcy and blood pressure (*P* < 0.05). Similarly, report from the third National Health and Nutrition Examination Survey (NHANES III) including 7,612 residents of USA aged 17 and above, showed that after adjusting for CVD risk factors, a 5 µmol/L increase in Hcy was associated with a 0.7 mmHg and 0.5 mmHg increase in systolic and diastolic BP in men and a 1.2 mmHg and 0.7 mmHg increase in systolic and diastolic BP in women [43]. Contrary to these, the Framingham Heart Survey, documented that after a median follow up of 2,104 subjects for 4 years, the age and sex-adjusted odds of hypertension incidence due to Hcy was negative and non-significant [38].

Positive association of Hcy with age or alcohol intake have been widely reported [14, 28, 31]. Though the mechanism underlying this association is vague, it is hypothesized that deficiency in folate and vitamin B12; metabolic changes, poor nutritional absorption and organ function associated with ageing or alcohol intake are possible causes of this anomaly [44–46].

In conclusion, HHcy occurs among adult Nigerians living with hypertension. Hcy associates independently with age, HDL-C, office systolic and diastolic BP. The inverse association of Hcy with HDL-C is clinically relevant considering the vital role that HDL-C plays to prevent against vascular diseases. In addition to this, HDL-C is understood to be crucially involved in the hydrolytic activity of paraoxonase-1 which leads to breakdown of Hcy-thiolactone (the Hcy derivative that is directly responsible for collagen damage) [47].

Our findings support the need for future research to conduct a pre-assessment of study population with a view to discovering the presence of interactive effects of Hcy and conventional risk factors on hypertension or CVD. This exercise is important as it is hypothesised that the presence of interactive effects of Hcy and other factors may exacerbate the association of Hcy with blood pressure, thus affecting how different populations respond to Hcy-lowering therapy.

Despite conflicting evidence regarding the CVD benefit of lowering plasma Hcy, which has hindered global adoption of Hcy guidelines in CVD care, routine assessment of Hcy in hypertensive subjects is necessary as emerging evidence [48] indicate that Hcy attenuates the efficacy of certain antihypertensive medications like angiotensin converting enzyme inhibitors. The therapeutic benefit of lowering Hcy remains a prospect if the PPARα-induced deregulation of apo-AI can be exploited because animal-based evidence indicates that certain PPARα agonists such as ciprofibrate improve endothelial function in mice with HHcy [49].

## Strengths and limitations

Given the small size of our sample, caution may be required when translating our findings to the general adult population of Nigeria. However, our subjects were drawn from a well-delineated area of Abuja, a cosmopolitan city with ethnically diverse population that has a semblance of the general Nigerian population. Our study was cross-sectional in design, hence the cause-effect association of Hcy with other factors could not be ascertained. Additionally, the renal function of the study participants which was not examined may have biased either the prevalence of HHcy or the association of Hcy with the selected risk factors of CVD.

## Supplementary Information

**Additional file 1.** Supplementary appendix REMAH Survey Questionnaire.

## Data Availability

The datasets used and/or analysed during the current study are available from the corresponding author on reasonable request.

## Abbreviations

AOR: Adjusted odds ratio
BMI: Body mass index
CBS: Cystathionine β-synthase
CI: Confidence interval
CVD: Cardiovascular diseases
DBP: Diastolic blood pressure
FCT-Abuja: Federal capital territory-Abuja
Hcy: Homocysteine
HDL-C: High-density lipoproteins Cholesterol
HHcy: Hyperhomocysteinemia
LCAT: Lecithin-cholesterol acyl transferase
LDL-C: Low-density lipoproteins Cholesterol
MTHFR: Methylene tetrahydrofolate reductase
OR: Odds ratio
PPARα: Peroxisome proliferator-activated receptor α
SBP: Systolic blood pressure
SREBPs: Sterol regulatory element binding proteins
REMAH: REmoving the MAsk on Hypertension
TAG: Triacylglycerol
NHANES III: National health and Nutrition examination survey III
VLDL: Very low-density lipoproteins
