# Supplementary material for: Hyperhomocysteinemia and its relations to conventional risk factors for cardiovascular diseases in adult Nigerians: the REMAH study
Source: BMC Cardiovasc Disord. 2021 Feb 18;21:102. doi: 10.1186/s12872-021-01913-x (PMC7890880; doi:10.1186/s12872-021-01913-x)
Supplement: Supplementary file 1 — Additional file 1. Supplementary appendix REMAH Survey Questionnaire. [file 12872_2021_1913_MOESM1_ESM.docx]

**Supplementary Files**

**Hyperhomocysteinemia and its Relations to Conventional Risk Factors for Cardiovascular Diseases in Adult Nigerians: The REMAH Study.**

Babangida S. Chori, Benjamin Danladi, Bassey A. Inyang, Michael P. Okoh, Maxwell M. Nwegbu, Adewale L. Alli, Augustine N. Odili

1. **Study Questionnaire Participant’s ID**

**REMOVING THE MASK ON HYPERTENSION**

**(REMAH) STUDY**

**PARTICIPANT’S QUESTIONNAIRE**

**Survey Information**

| **LOCATION AND DATE** | | **RESPONSE** | **CODE** |
| --- | --- | --- | --- |
| 1 | Site ID |  | 11 |
| 2 | Site name |  | 12 |
| 3 | Interviewer ID |  | 13 |
| 4 | Date of completion of the instrument | dd mm year | 14 |
| **Consent, Interview Language and Name** | | **Response** | **Code** |
| 5 | Consent has been read and obtained | Yes 1  No 2 If NO, END | 15 |
| 6 | Interview Language (Insert Language) | English 1  Hausa 2  Igbo 3  Yoruba 4  Others: ............................................. | 16 |
| 7 | Time of interview  (24 hour clock) | hrs mins | 17 |
| 8 | Family Surname |  | 18 |
| 9 | First Name |  | 19 |
| 10 | Contact phone number where possible |  | 110 |
| 11 | Contact Email address |  | 111 |

**Step 1 Demography Information**

| **CORE: Demography Information** | | | |
| --- | --- | --- | --- |
| **Question** | | **Response** | **Code** |
| 12 | Sex (Record Male/Female as observed) | Male 1  Female 2 | C1 |
| 13 | What is your date of birth?  Don’t know 77 77 7777 | If known, Go to C4  dd mm year | C2 |
| 14 | How old are you? | Years | C3 |
| 15 | What is the highest level of education you have completed? | No formal schooling 1  Less that primary school 2  Primary school completed 3  Secondary school completed 4  COE/Poly/Uni completed 5  Post graduate degree 7  Refused 88 | C4 |
| 16 | What is your ethnic background? | Father’s tribe ………………………….  Mother’s tribe ………………………..  Refused 88 | C5 |
| 17 | What is your marital status? | Never married 1  Currently married 2  Separated 3  Divorced 4  Widowed 5  Cohabitating 6  Refused 88 | C6 |
| 18 | Which of the following best describe your main work status over the past 12 months? | Government employee 1  Non-government employee 2  Self-employed 3  Non-paid 4  Student 5  Housewife 6  Retired 7  Unemployed (able to work) 8  Unemployed (unable to work) 9  Refused 88 | C7 |
| 19 | How many people older than 18 years, including yourself, live in your household? | Number of people | C8 |

**Step 1 Behavioural Measurements**

| **CORE: Tobacco Use** | | | |
| --- | --- | --- | --- |
| Now I am going to ask you some questions about various health behaviours. This includes things like smoking, drinking alcohol, eating fruits and vegetables and physical activity. Let’s start with tobacco. | | | |
| **Question** | | **Response** | **Code** |
| 20 | Do you currently smoke any tobacco products, such as cigarettes, cigars or pipes? | Yes 1  No 2 If No, go to T6 | T1 |
| 21 | Do you currently smoke tobacco product daily? | Yes 1  No 2 If No, go to T6 | T2 |
| 22 | How old where you when you first started smoking daily? | Age (year)  Don’t know 77 if no, go to T5a | T3 |
| 23 | Do you remember how long ago it was?  (RECORD ONLY 1, NOT ALL 3)  Don’t know 77 | In Years If no, go to T5a | T4a |
|  |  | OR in months if no, go to T5a | T4b |
|  |  | OR in weeks | T4c |
| 24 | On average, how many of the following do you smoke each day?  Don’t know 77 | Manufactured cigarettes | T5a |
|  |  | Hand-rolled cigarettes | T5b |
|  |  | Pipes full of tobacco | T5c |
|  |  | Cigars | T5d |
|  |  | Other if other, go to T5other,  else go to T9 | T5e |
|  |  | OTHER (PLEASE SPECIFY)  If no, go to T9 | T5other |
| 25 | In the past, did you ever smoke daily? | Yes 1  No 2 if No, go to T9 | T6 |
| 26 | How old were you when you stopped smoking daily? | Age (years)  Don’t know 77 if no, go to T9 | T7 |
| 27 | How long ago did you stop smoking daily?  (record only 1, not all 3)  Don’t know 77 | Years ago if no, go to T9 | T8a |
|  |  | OR months ago if no, go to T9 | T8b |
|  |  | OR weeks ago | T8c |
| 28 | Do you currently use any smokeless tobacco such as (snuff, chewing tobacco, betel)? | Yes 1  No 2 if No, go to T12 | T9 |
| 29 | On average, how many times a day do you use… | Snuff, by mouth | T10a |
|  |  | Snuff, by nose | T10b |
|  |  | Chewing tobacco | T10c |
|  |  | Don’t know 77 | T10d |
|  |  | Other if other, go to T11other, else go to T12 | T10e |
| 30 | During the past 7 days. On how many days did someone in the home smoke when you were present? | Number of days  Don’t know 77 | T11 |
| 31 | During the past 7 days, on how many days did someone smoke in closed areas in your workplace or relaxation centres (in the building, in a work area or a specific office) when you were present? | Number of days  Don’t know or don’t  work in a closed area 77 | T12 |
| **CORE: Alcohol Consumption** | | | |
| **The next questions ask about the consumption of alcohol.** | | | |
| **Question** | | **Response** | **Code** |
| 32 | Have you ever consumed an alcoholic drink such as beer, palm wine, red wine, spirits, and fermented drinks? | Yes 1  No 2 if No, go to P1 | A1a |
| 33 | Have you consumed an alcoholic drink within the past 12 months? | Yes 1  No 2 if No, go to P1 | A1b |
| 34 | During the past 12 months, how frequently have you had at least one alcoholic drink? | Daily 1  5-6 days per week 2  1-4 days per month 3  1-3 days per week 4  Less than once a month 5 | A2 |
| 35 | Have you consumed an alcoholic drink within the past 30 days? | Yes 1  No 2 | A3 |
| **CORE: Physical Activity** | | | |
| Next I am going to ask you about the time you spend doing different types of physical activity in a typical week. Please answer these questions even if you do not consider yourself to be a physically active person.  Think first about the time you spend doing work. Think of work as the things that you have to do such as paid or unpaid work, study/training, household chores, harvesting food/crops, fishing or hunting for food, seeking employment. [Insert other examples if needed]. In answering the following questions ‘vigorous-intensity activities’ are activities that require hard physical effort and cause large increases in breathing or heart rate, ‘moderate-intensity activities’ are activities that require moderate physical effort and cause small increases in breathing or heart rate. | | | |
| **Question** | | **Response** | **Code** |
| **Work** | | | |
| 36 | Does your work involve vigorous-intensity that causes large increases in breathing or heart rate like (carrying or lifting heavy loads, digging or construction work) for at least 10 minutes continuously? | Yes 1  No 2 if No, go to P4 | P1 |
| 37 | In a typical week, on how many days do you do vigorous-intensity activities as part of your work? | Number of days | P2 |
| 38 | How much time do you spend doing vigorous-intensity activities at work on a typical day? | Hours: minutes  hrs mins | P3(a-b) |
| 39 | Does your work involve moderate-intensity activity that causes small increases in breathing or heart rate such as brisk walking [or carrying light loads] for at least 10 minutes continuously? | Yes 1  No 2 if No, go to P7 | P4 |
| 40 | In a typical week, on how many days do you do moderate-intensity activities as part of your work? | Number of days | P5 |
| 41 | How much time do you spend doing moderate-intensity activities at work or a typical day? | Hours: minutes  hrs mins | P6(a-b) |
| **Travel to and from places** | | | |
| The next questions exclude the physical activities at work that you have already mentioned.  Now I would like to ask you about the usual way you travel to and from places. For example to work, for shopping, to market, to place of worship. [Insert other examples if needed]. | | | |
| 42 | Do you walk or use a bicycle (pedal cycle) for at least 10 minutes continuously to get to and from places? | Yes 1  No 2 if No, go to P10 | P7 |
| 43 | In a typical week, on how many days do you walk or bicycle for at least 10 minutes continuously to get to and from places? | Number of days | P8 |
| 44 | How much time do you spend walking or bicycling for travel on a typical day? | Hours: minutes  hrs mins | P9(a-b) |
| **CORE: Physical Activity, Continued** | | | |
| **Question** | | **Response** | **Code** |
| **Recreational activities** | | | |
| The next questions exclude the work and transport activities that you have already mentioned.  Now I would like to ask you about sports, fitness and recreational activities (leisure), [Insert relevant terms]. | | | |
| 45 | Do you do any vigorous-intensity sports, fitness or recreational (leisure) activities that cause large increases in breathing or heart rate like [running or football] for at least 10 minutes continuously? | Yes 1  No 2 if No, go to P13 | P10 |
| 46 | In a typical week, on how many days do you do vigorous-intensity sports, fitness or recreational (leisure) activities? | Number of days | P11 |
| 47 | How much time do you spend doing vigorous-intensity sports, fitness or recreational activities on a typical day? | Hours: minutes  hrs mins | P12(a-b) |
| 48 | Do you do any moderate-intensity sports, fitness or recreational (leisure) activities that cause a small increase in breathing or heart rate such as brisk walking, (cycling, swimming, and volleyball) for at least 10 minutes continuously? | Yes 1  No 2 if No, go to P16 | P13 |
| 49 | In a typical week, on how many days do you do moderate-intensity sports, fitness or recreational (leisure) activities? | Number of days | P14 |
| 50 | How much time to you spend doing moderate-intensity sports, fitness or recreational (leisure) activities on a typical day? | Hours: minutes  hrs mins | P15(a-b) |
| **CORE: History of Raised Blood Pressure** | | | |
| **Question** | | **Response** | **Code** |
| 51 | Have you ever had your blood pressure measured by a doctor or other health worker? | Yes 1  No 2 if No, go to H6 | H1 |
| 52 | Have you ever been told by a doctor or other health worker that you have raised blood pressure or hypertension? | Yes 1  No 2 if No, go to H6 | H2 |
| **Expanded: History of Raised Blood Pressure** | | | |
| 53 | Are you currently receiving any of the following treatments/advice for high pressure prescribed by a doctor or other health worker? | | |
|  | Drugs (medication) that you have taken in the past two weeks | Yes 1  No 2 | H3a |
|  | Advice to reduce salt intake | Yes 1  No 2 | H3b |
|  | Advice or treatment to lose weight | Yes 1  No 2 | H3c |
|  | Advice or treatment to stop smoking | Yes 1  No 2 | H3d |
|  | Advice to start or do more exercise | Yes 1  No 2 | H3e |
| 54 | Have you ever seen a traditional healer for raised blood pressure or hypertension? | Yes 1  No 2 | H4 |
| 55 | Are you currently taking any herbal or traditional remedy for your raised blood pressure? | Yes 1  No 2 | H5 |
| **CORE: History of Diabetes** | | | |
| **Question** | | **Response** | **Code** |
| 56 | Have you ever had your blood sugar measured by a doctor or other health worker? | Yes 1  No 2 if No, go to H6 | H6 |
| 57 | Have you ever been told by a doctor or other health worker that you have raised blood sugar or diabetes? | Yes 1  No 2 if No, go to H6 | H7 |
| 58 | Are you currently receiving any of the following treatments/advice for high blood sugar prescribed by a doctor or other health worker? | | |
|  | Insulin | Yes 1  No 2 | H8a |
|  | Drugs (medication) that you have taken in the past two weeks | Yes 1  No 2 | H8b |
|  | Special prescribed diet | Yes 1  No 2 | H8c |
|  | Advice or treatment to lose weight | Yes 1  No 2 | H8d |
|  | Advice or treatment to stop smoking | Yes 1  No 2 | H8e |
|  | Advice to start or do more exercise | Yes 1  No 2 | H8f |
| 59 | Have you ever seen a traditional healer for diabetes or raised blood sugar? | Yes 1  No 2 | H9 |
| 60 | Are you currently taking any herbal or traditional remedy for your diabetes? | Yes 1  No 2 | H10 |

**Step 2 Physical Measurements**

| **CORE: Height and Weight** | | | |
| --- | --- | --- | --- |
| **Question** | | **Response** | **Code** |
| 61 | Interviewer ID |  | M1 |
| 62 | Cuff size | Small 1 Medium 2 Large 3 | M2 |
| 63 | Arm circumference |  | M3 |
| 64 | Device ID for blood pressure |  | M4 |
| 65 | Reading 1 | SBP | M5a |
|  |  | DBP | M5b |
| 66 | Reading 2 | SBP | M6a |
|  |  | DBP | M6b |
| 67 | Reading 3 | SBP | M7a |
|  |  | DBP | M7b |
| 68 | Reading 4 | SBP | M8a |
|  |  | DBP | M8b |
| 69 | Reading 5 | SBP | M9a |
|  |  | DBP | M9b |
| 70 | Pulse Rate |  | M10 |
| 71 | Height |  | M11 |
| 72 | Weight |  | M12 |
| 73 | Waist circumference |  | M13 |
| 74 | Hip circumference |  | M14 |
| 75 | ECG |  | M15 |
| **Sample Collected** | | | |
| 76 | Interviewer’s ID |  | B1 |
| 77 | When did you take your last meal (including snacks), other than water? | Hrs | B2 |
| 78 | Time of day blood specimen taken | Hrs mins | B3 |
| 79 | Blood dropped on filter paper | Yes | B4 |
| 80 | Date of 24 hour urine Collection | dd mm year | B5 |
| 81 | Volume of urine |  | B6 |
| 82 | **Home BP**   \|  \| **AM1** \| \| \| **AM2** \| \| \| **PM1** \| \| \| **PM2** \| \| \| \| --- \| --- \| --- \| --- \| --- \| --- \| --- \| --- \| --- \| --- \| --- \| --- \| --- \| \| **DAYS** \| **SBP** \| **DBP** \| **PR** \| **SBP** \| **DBP** \| **PR** \| **SBP** \| **DBP** \| **PR** \| **SBP** \| **DBP** \| **PR** \| \| **1** \|  \|  \|  \|  \|  \|  \|  \|  \|  \|  \|  \|  \| \| **2** \|  \|  \|  \|  \|  \|  \|  \|  \|  \|  \|  \|  \| \| **3** \|  \|  \|  \|  \|  \|  \|  \|  \|  \|  \|  \|  \| | | B7 |
| 83 | ABPM | Uploaded | B8 |
